# c‐Myb facilitates immune escape of esophageal adenocarcinoma cells through the miR‐145‐5p/SPOP/PD‐L1 axis

**DOI:** 10.1002/ctm2.464

**Published:** 2021-09-26

**Authors:** Lan Zhang, Xiaohui Wang, Yunfei Li, Jing Han, Xianzheng Gao, Shenglei Li, Feng Wang

**Affiliations:** ^1^ Department of Pathology The First Affiliated Hospital of Zhengzhou University Zhengzhou P. R. China; ^2^ Department of Pathology The First Affiliated Hospital of Xinxiang Medical University Zhengzhou P. R. China; ^3^ Department of Oncology The First Affiliated Hospital of Zhengzhou University Zhengzhou P. R. China

**Keywords:** c‐Myb, esophageal adenocarcinoma, immune escape, microRNA‐145‐5p, programmed death ligand 1, speckled POZ protein

## Abstract

Esophageal adenocarcinoma (EAC), a subtype of esophageal carcinoma, is a severe health problem associated with high death rate and poor prognosis. Immunotherapy has proven to be effective in many solid tumors, including EAC, but immune escape blocks its effectiveness. Thus, we explored the mechanisms and functional role of c‐Myb in immune escape of EAC cells. Clinical EAC tissues were collected for determining the expression of c‐Myb, speckled POZ protein (SPOP), and miR‐145‐5p. Functional assays were then performed to detect the interactions between c‐Myb and SPOP as well as between SPOP and miR‐145‐5p. EAC cell invasion and migration were assessed. Next, T cells were sorted and cocultured with EAC cells with different treatments followed by detection of T‐cell viability. In addition, a mouse model of EAC was constructed for relevant in vivo assays. c‐Myb and miR‐145‐5p were highly expressed and SPOP had low expressions in EAC. c‐Myb activated the transcription of miR‐145‐5p, which in turn targeted SPOP. Further, SPOP accelerated the ubiquitination of PD‐L1 to enhance its expression. Overexpression of PD‐L1 suppressed T‐cell functions and promoted proliferative and migrative abilities of EAC cells to induce immune escape. The above findings were also confirmed in the ECA mouse model in vivo. Our findings uncovered that c‐Myb can promote the immune escape of EAC cells by favoring the transcription of miR‐145‐5p and inhibiting SPOP‐dependent ubiquitination and degradation of PD‐L1, thus, presenting new target for EAC adjunct therapy.

## 1 INTRODUCTION

Esophageal adenocarcinoma (EAC) is known as an aggressive malignancy with high mortality.^1^ Gastroesophageal reflux disease, obesity, tobacco smoking, and Barrett's esophagus are risk factors for EAC.^2^ Among these factors, Barrett's esophagus is deemed to be the precursor lesion of this neoplasm.^3^ EAC is characterized by male predominance, increasing incidence globally, lack of methods for early detection, and poor prognosis.^4^ Recently, immunotherapy has been developed as an innovative, promising treatment measure for many solid tumors including EAC.^5^ However, immune escape is implicated in cancer progression, and tumors express multiple mechanisms to stimulate their immune escape, which remains a primary barrier to successful immunotherapy for cancers.^6^, ^7^ In order to enhance the immunotherapeutic efficacy for EAC, we explored in this article potential immune escape mechanisms of EAC cells.

Using bioinformatics tools, we first identified MYB as a differentially expressed gene associated with EAC. The c‐Myb gene encodes a transcription factor and is often overexpressed in human solid tumors, implicated it in cell differentiation, proliferation, and apoptosis via protein‐protein interactions and signaling pathway transcriptional regulation.^8^, ^9^ In particular, c‐Myb is highly expressed in EAC,^10^ implying it to be a contributing factor in EAC. Moreover, c‐Myb overexpression (OE) is involved in esophageal carcinoma as it is upregulated in esophageal carcinoma tissues and its high expression predicts poor prognosis.^11^ In addition, the proto‐oncogene MYB has a promotive effect on the transcription of miR‐145‐5p,^12^ which is highly expressed in EAC and accelerates EAC progression by raising cell invasion and anoikis resistance.^13^ Thus, c‐Myb may regulate miR‐145‐5p to facilitate EAC development and promote anchorage to the extracellular matrix. Furthermore, miR‐145 could downregulate speckled POZ protein (SPOP), a regulator of the ubiquitination process based on Cullin 3^14^ that is altered in a variety of types of cancer and potentially exerts a pleiotropic tumorigenic effect, given that multiple regulatory signaling pathways are dysregulated upon SPOP alteration.^15^ On this basis, miR‐145‐5p may reduce SPOP to affect the development of EAC. Previous research also shows that SPOP can promote the ubiquitination modification of serum programmed death ligand 1 (PD‐L1) to inhibit tumor development.^16^ PD‐L1 is the ligand of PD‐1, which is a coinhibitory receptor mainly expressed by T cells, and expression of which is an independent indicator of good outcomes in patients with localized EAC.^17^ Meanwhile, PD‐L1 has been reported to be associated with weakened anticancer immunity.^18^ Moreover, tumor cells can lead to immune escape by inhibiting the function of T cells through regulating PD‐L1.^19^ Based on the above bioinformatics analysis and literature reports, we hypothesize that a novel c‐Myb/miR‐145‐5p/SPOP/PD‐L1 axis functions in the immune escape mechanism of EAC cells.

**Graphical Highlights**

C‐Myb is upregulated in EAC and indicates poor prognosis of patients with EAC.

C‐Myb promotes transcription of miR‐145‐5p in EAC cells.

miR‐145‐5p targets SPOP to enhance immune escape of EAC cells.

SPOP facilitates ubiquitination and degradation of PD‐L1 in EAC cells.

## 2 Materials and METHODS

### 2.1 Tissue sample collection

EAC tissues were collected from 30 patients admitted into the Digestive Surgery Department from December 2011 to December 2012. The clinical data of patients were summarized, and all were pathologically diagnosed as having primary EAC. Meanwhile, tumor adjacent tissues were collected during the EAC resection, which served as normal control material upon pathological verification of the absence of tumor cell infiltration in the tissues. All collected samples were stored at −80°C.

### 2.2 Follow‐up of EAC patients after resection

Follow‐up of EAC patients was conducted from 3 months after curative resection of EAC until the patients died or loss to follow‐up, with recording of the average follow‐up duration. Postoperative re‐examination of patients was performed once every 3 months for 2 years, once every 6 months during 3‐5 years, and once every year after 5 years following the resection. The re‐examination items included routine blood chemistry and cytology, gastroscopy, chest CT (plain scan or enhanced scan), and neck + abdomen B ultrasound examination. Physical examination was also required based on their symptoms, accompanied by imaging examinations such as PET‐CT, bone scanning, and MRI. Finally, postoperative survival of the patients was calculated based on follow‐up data.

### 2.3 IHC

EAC tissues (4‐μm‐thick) were processed routinely for IHC staining. The primary antibodies used in IHC comprised of c‐Myb (1:500, bs‐5978R, Beijing Bioss Biotechnology Co. Ltd., Beijing, China), SPOP (1:500, 16750‐1‐AP, Proteintech Group, Wuhan, Hubei, China), CD3 (1:500, 17617‐1‐AP, Proteintech Group), and Granzyme B (1:500, 13588‐1‐AP, Proteintech Group). Images of the sections were photographed via Leica Microsystems (DM2000, Germany). IHC‐positive cells were counted by ImagePro Plus 6.0 (Media Cybernetics).

### 2.4 RT‐qPCR

TRIzol (15596026, Invitrogen, Carlsbad, CA) was used to extract total RNA from the cells. The mRNA and miRNA were reversely transcribed into cDNA. The synthesized cDNA was prepared with RT‐qPCR using Fast SYBR Green PCR kit (Applied Biosystems, CA) and an ABI PRISM 7300 QPCR system (Applied Biosystems). GAPDH served as mRNA control, and U6 as miRNA control. The gene expression was analyzed using the 2^−ΔΔ^ *^Ct^* method. The used primers are shown in Supporting Information Table S1.

### 2.5 Cell culture and transfection

Human EAC cell lines OE33 and FLO‐1 as well as human normal esophageal epithelial cell line HET‐1A from ATCC were cultured in RPMI‐1640 (Gibco, Carlsbad, CA). The two types of cells were cocultured at 1:1. The cells were detached and seeded into 12‐well plates at 8 × 10^4^ cells/well. After 24 h of routine culture to obtain confluence of about 75%, lentiviral transfection was conducted according to kit instructions. First, cell lines overexpressing or underexpressing c‐Myb were screened out and transfected with short hairpin RNA‐NC (sh‐NC), sh‐c‐Myb, vector, c‐Myb OE, mimic NC, miR‐145‐5p mimic, inhibitor NC, miR‐145‐5p inhibitor, sh‐SPOP, SPOP OE, and shCullin3. The related primers are shown in Supporting information Table S2.

### 2.6 Preparation of CD8^+^ T cells and flow cytometry

Human peripheral blood lymphocytes were purchased from the Biotherapy Center of Zhongnan Hospital of Wuhan University. PerCP‐CD3 antibody and PE‐CD8 antibody were mixed with lymphocytes cultured in vitro and incubated at 4°C for 10 min. PerCP and PE‐labeled mouse anti‐human IgG were used as an isotype control. The composition of lymphocytes was detected by flow cytometry. Human lymphocytes cultured in vitro were resuspended with 100 μL cell buffer and then sorted by magnetic beads for CD8^+^ T cells. Briefly, CD8 antibody magnetic beads were added for 15 min incubation at 4°C. After resuspension, the cells were added into the adsorption column installed on a magnetic base, finally obtaining CD8^+^ T cells. Then, the purity of the sorted CD8^+^ T cells was detected on the flow cytometer using the detection step identical to the above for determining lymphocyte composition. PE‐labeled monoclonal antibody of PD‐1 was reacted with CD8^+^ T cells sorted by magnetic beads at 4°C for 10 min in the dark, and PE‐labeled mouse anti‐human IgG was used as an isotype control to detect PD‐1 expression.

### 2.7 Dual luciferase reporter gene assay

The procedure was performed according to instructions of Promega fluorescence dual reporter system. We constructed fluorescent plasmids by inserting wild‐type (WT) and mutant (MUT) 3′‐UTR of SPOP into pGL3.0‐basic vector. The fluorescent plasmid was divided into two equal parts. One part was directly transfected into EAC cells (control group), and the other part was cotransfected with miR‐145‐5p mimic lentivirus into EAC cells. After 48 h of transfection, luciferase activity was measured.

### 2.8 Detection of Th1 and Th2 cytokines

Six cytokines secreted by Th1 and Th2 cells, including interleukin (IL)‐2, IL‐4, IL‐5, IL‐10, tumor necrosis factor (TNF)‐a, and interferon (IFN)‐g, were detected using CBA Th1/Th2 cytokine kits (BD Cytometric Bead Array) according to the instructions. Th1 cytokine level was expressed as concentrations of IFN‐g, IL‐2, and TNF‐a, and Th2 cytokine levels were expressed as concentrations of IL‐4, IL‐5, and IL‐10. Standard curves were drawn by BD CellQuest software BD CBA (BD Bioscience) and the concentrations of sample cytokines were calculated.

### 2.9 Cell proliferation detection

The T cells cocultured with FLO‐1 cells following different treatments were selected. T cells were labeled with 0.5 μM carboxyfluorescein succinimidyl ester (Life Technologies, Carlsbad, CA). After accurate counting, the T cells were seeded in a 96‐well plate after, and 200 μL cell suspension with a total of 3000 cells was added to each well. After adhered, cells were stained by fluorochromeconjugated antibodies (BD Biosciences). After incubated for 2 h, the fluorescence changes were detected by flow cytometry.

### 2.10 Cell apoptosis detection

A total of 100 μL cell suspension was taken and tested according to the experimental method outlined in the Muse^®^ Annexin V & Dead Cell Kit (Millipore, Billerica, MA), and then detected by flow cytometry. AnnexinV was replaced by CD3 antibody during the process.

### 2.11 Transwell assay

Cell invasion and migration activity was analyzed using Transwell chamber coated with/without Matrigel (BD Biosciences). The FLO‐1 cells cocultured with T cells were resuspended and seeded into the apical chamber, while 10% FBS was added to basolateral chamber. After 24 h, cells were fixed with 100% methyl alcohol and then stained with 1% toluidine blue (Sigma‐Aldrich Chemical Company, St. Louis, MO). The cells passed through the membrane were counted by an inverted light microscope (CarlZeiss, Oberkochen, Germany).

### 2.12 Western blot analysis

The extracted protein was separated with 10% SDS‐PAGE and electrotransferred. After blocking, the membrane was incubated with GAPDH (1:5000, ab181602), E‐cadherin (1:1500, ab3195), N‐cadherin (1:1500, ab13116), MMP3 (1:1500, ab14351), MMP9 (1:1500, ab13667), FasL (1:1500, ab15285) from Abcam Cambridge, UK as well as antibodies to SPOP (1:1000, 16750‐1‐AP), PD‐L1 (1:1000, 28076‐1‐AP), Vinculin (1:1000, 26520‐1‐AP), and Fas (1:1000, 13098‐1‐AP) from Proteintech Group overnight at 4°C. Then the membrane was incubated with secondary antibody (ab205719, 1:2000, Abcam) for 1 h and added with enhanced chemiluminescence working fluid (Millipore) for coloring. Finally, Image J software was employed to quantify the gray levels.

### 2.13 ELISA

The human IFN‐γ (ab174443), mouse IFN‐γ (ab100689), human IL‐2 (ab46054), mouse IL‐2 (ab47591), human IL‐4 (ab215089), mouse IL‐4 (ab100710), human IL‐10 (ab185986), and mouse IL‐10 (ab255729) ELISA kits (Abcam) were used.

### 2.14 RIP

RIP kit (Millipore) was used to detect binding of related RNAs to protein. Cells were lysed for 5 min and centrifuged at 14 000 rpm and 4°C for 10 min to obtain the supernatant. A portion of cell extract was taken as input, and the left part was incubated with antibody for coprecipitation.

RNA was extracted after proteinase K digestion. The dilution concentrations of the antibodies used for RIP were as follows: SPOP (10 μL, 16750‐1‐AP) and IgG (10 μL, 10285‐1‐AP), and the antibodies were produced by Proteintech group. The antibodies were mixed, with IgG serving as negative control.

### 2.15 RNA pull‐down assay

A Pierce Magnetic RNA pull‐down kit (20,164, Thermo Fisher Scientific, Waltham, MA) was used. miR‐145‐5p RNA was transcribed using a DNA template by Ribo RNAmax‐T7 transcription kit (RiboBio, Guangzhou, China). miR‐145‐5p was labeled with desulfobiotin using the Pierce RNA 3′‐end desulfobiotinylation kit (20,163, Thermo Fisher Scientific) and added to EAC cells. Biotinylated miR‐145‐5p was captured and mixed with EAC cell extracts. Then protein was eluted and detected using SPOP antibody.

### 2.16 Xenograft tumors in mice

Forty BALB/c mice aged 5 weeks (Beijing Institute of Pharmacology, Chinese Academy of Medical Sciences, Beijing, China) were used. Mice were grouped as (1) sh‐NC group; (2) sh‐NC + PD‐L1 Ab group; (3) sh‐c‐Myb group; and (4) sh‐c‐Myb + PD‐L1 Ab group (10 mice in each group). The mice were routinely housed. The experiment was started after adaptive feeding for 1 week. During feeding, 2 × 10^6^ cells per side, EAC cells were injected into the axillae of both upper limbs at a number, and the size and weight of the tumor mass were measured twice a week for 6 weeks according to formula (length × width^2^)/2. After 6 weeks, mice were euthanized by anesthesia overdose and tumor size and weight were measured.

### 2.17 Detection of Th1 and Th2 by flow cytometry

Mouse blood was drawn and heparinized, and the blood was incubated with the following fluorescent antibodies: phycocyanin/flower 7‐conjugated anti‐mouse CD4, (#100414, BioLegend Inc., San Diego, CA); phycoerythrin‐conjugated anti‐mouse CCR5, (#107006, BioLegend Inc.); sphingomyelin chlorophyll protein/cyanine 5.5‐conjugated anti‐mouse CXCR3 (#126514, BioLegend Inc.). Fluorescein and isothiocyanate‐conjugated anti‐CCR8, #ABIN732870, was provided by General Electric Co. Ltd. (Atlanta). Subsequently, the red blood cells were lysed by BD FACS ™ Lysis Solution (349202, BD Biosciences), and the samples were collected using a BD FACS Canto II (BD Biosciences). Data were assessed using FlowJo (TreeStar Inc., Ashland, OR). First, the lymphocyte population was gated and then CD4^+^ T cells were selected for further detailed analysis of the presence of CXCR3, CCR5, and CCR8 chemokine receptors. Based on the typical expression of chemokine receptors in these cells, CXCR3^+^ and CCR5^+^ cells were regarded as Th1 and CCR8^+^ cells as Th2.

### 2.18 Statistical analysis

Measurement data were expressed as mean ± standard deviation. Unpaired *t*‐test was used in intergroup comparisons. One‐way ANOVA or two‐way ANOVA was used for multiple group comparison with Tukey's post‐hoc test. The survival rate was calculated using the Kaplan–Meier method, and univariate analysis was performed with the log‐rank test. Pearson correlation was used to analyze the correlation of indicators. *P* value < .05 was indicative of significant statistical difference.

## 3 RESULTS

### 3.1 SPOP is poorly expressed in EAC and may be regulated by miR‐145‐5p and c‐Myb

**FIGURE 1:**
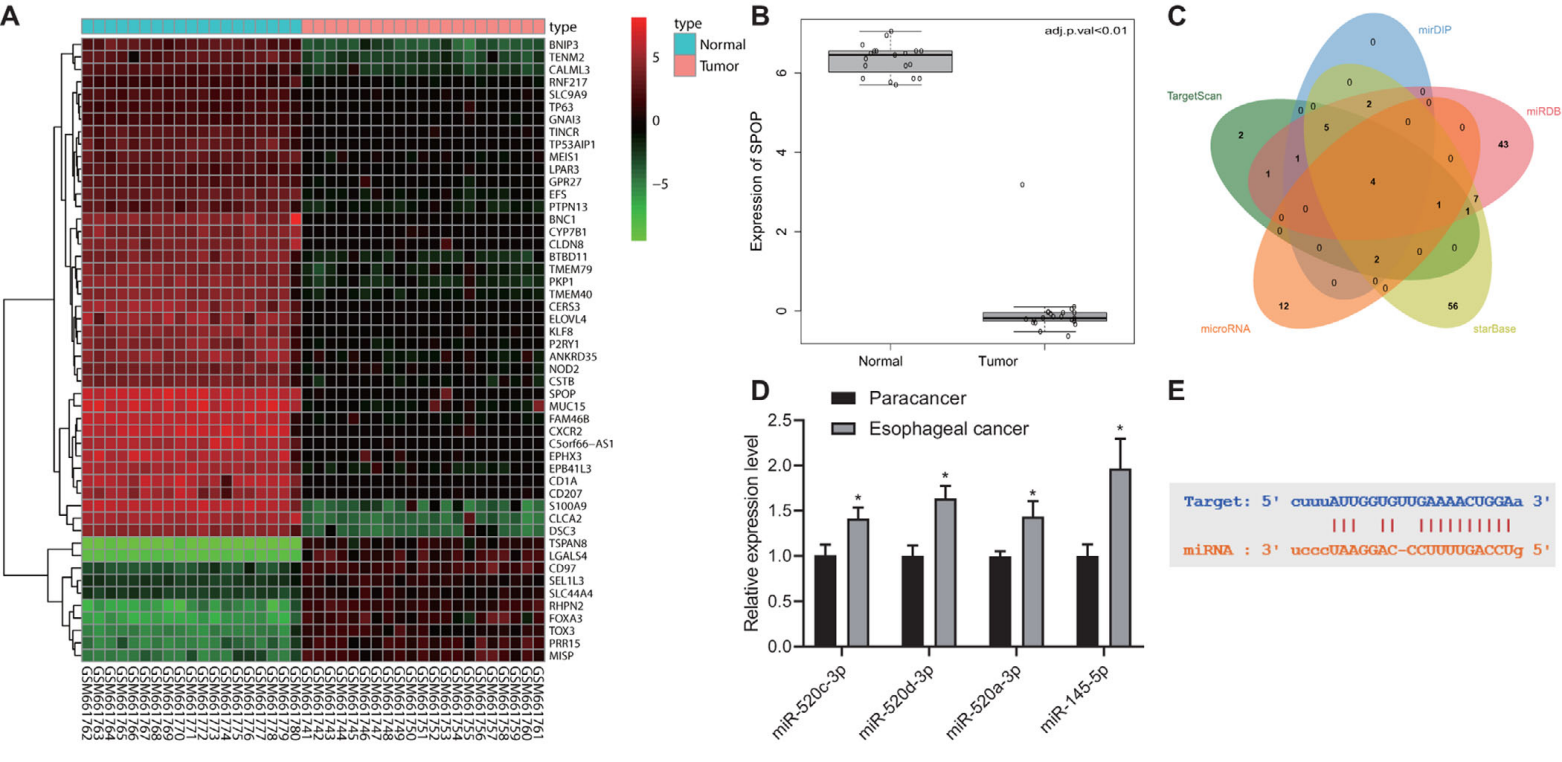
Expression of SPOP detection and its upstream miRNAs prediction in EAC. (A) The differentially expressed gene expression thermogram of GSE26886 in EAC datasets. In the heat map, the abscissa represents the sample number, the ordinate represents the gene name, each small square represents the expression of a gene in a sample, and the upper right histogram is the color scale. (B) Differential expression of SPOP in GSE26886. The box graph on the left shows normal samples, the box graph on the right shows tumor samples, and the upper right corner shows the differential *p* value. (C) The upstream miRNA prediction of SPOP. The four ellipses in the figure represent the prediction results of databases such as TargetScan, and the middle part represents the intersection of the four groups of data. (D) RT‐qPCR used to quantitatively analyze the expression levels of candidate miRNAs in EAC and adjacent tissues (n = 30). (E) miR‐145‐5p and SPOP target binding site information. *P < .05

Figure 1A shows the top 50 differentially expressed gene expression with heat map. Among these genes, SPOP in EAC was downregulated (Figure 1B). In addition, SPOP can affect tumor through the ubiquitination regulation of PD‐L1,^16^ and PD‐L1 is a marker gene of EAC.^20^ Then, upstream miRNAs of SPOP were further predicted by TargetScan and other databases, and intersection was obtained (Figure 1C). Finally, four candidate miRNAs were obtained (Supporting information Table S3). Furthermore, we detected expression of these four miRNAs in clinical tumor tissues. Expression of these four miRNAs were upregulated in EAC tissues, but change of miR‐145‐5p expression was the most significant (Figure 1D), and the StarBase database showed that there was a binding site between SPOP and miR‐145‐5p (Figure 1E). In addition, C‐Myb can regulate the progress of disease by promoting the transcription of miR‐145‐5p.^12^ JASPAR database predicted that there were multiple MYB transcription factor binding structures in promoter region of miR‐145‐5p (Supporting information Table S4).

### 3.2 c‐Myb and miR‐145‐5p are highly expressed in EAC

**FIGURE 2:**
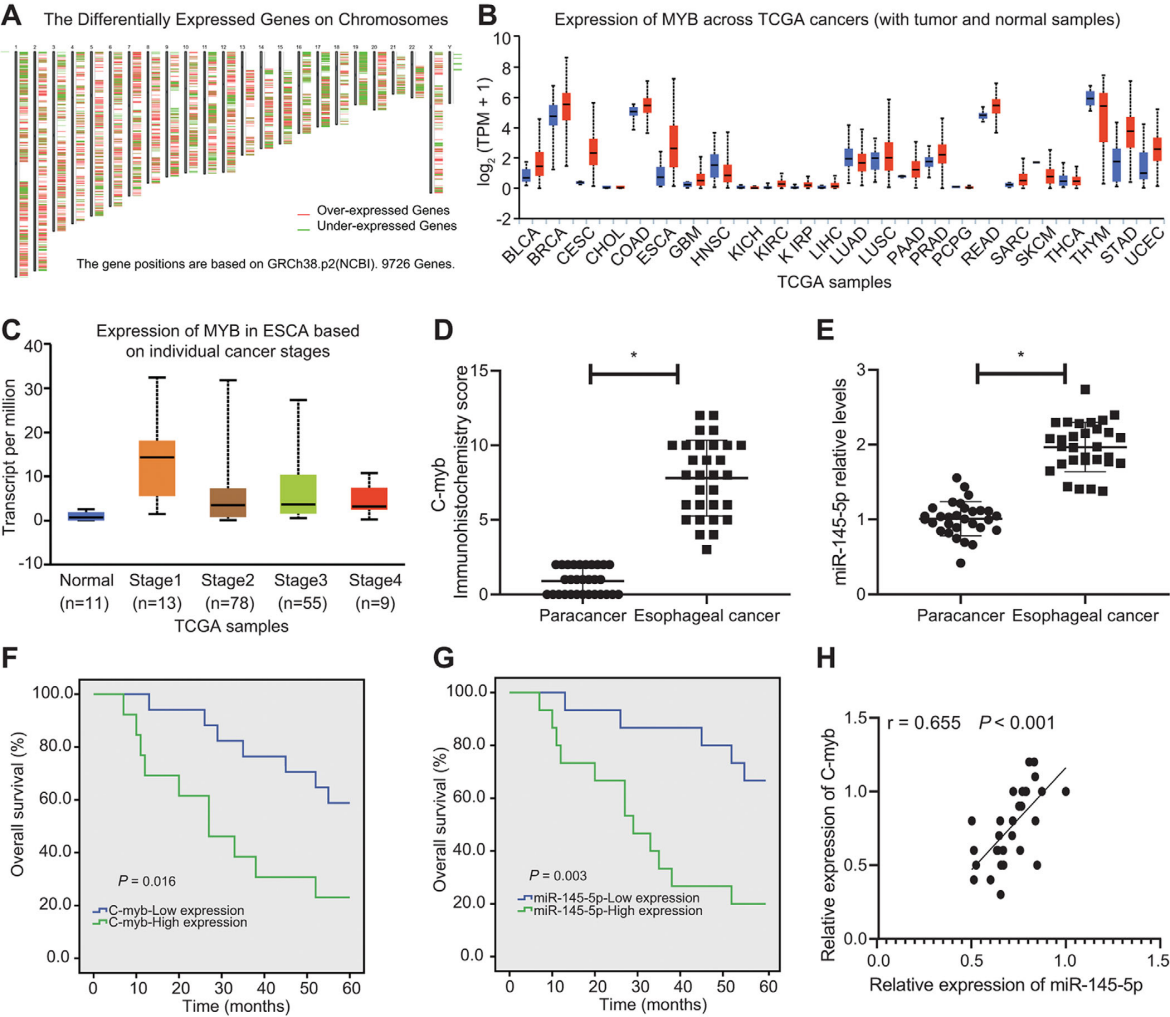
c‐Myb and miR‐145‐5p are highly expressed in EAC, and are associated with dismal prognosis. (A) Heat maps of expression of EAC‐related DEGs in TCGA, wherein the upper abscissa represents the number of chromosomes, red represents highly expressed genes, and green represents lowly expressed genes; each line represents a gene, the position of which represents the position of this gene in the chromosome. (B) The expression of c‐Myb in TCGA, wherein the abscissa represents the type of disease, the red boxes represent EAC samples, and the blue boxes represent normal samples. (C) The expression of c‐Myb in EAC of different stages, wherein the abscissa represents the sample stage, and the ordinate represents the expression value; the blue box plot on the left represents the c‐Myb expression in normal samples, and the other four box plots represent EAC of different disease stages. (D) IHC scoring of c‐Myb in clinical samples (n = 30). (E) RT‐qPCR determination of miR‐145‐5p expression in EAC samples and tumor adjacent samples (n = 30). (F) Regression analysis of the correlation of c‐Myb expression with the total survival of EAC patients. (G) Regression analysis of the correlation of miR‐145‐5p expression with the total survival of EAC patients. (H) Analysis of the correlation of c‐Myb expression with miR‐145‐5p expression in EAC samples. *P < 0.05. Measurement data were expressed as the mean ± standard deviation. Paired *t‐*test was used for data analysis in panels E and F, Kaplan–Meier method was used to calculate survival rate of patients followed by one‐way ANOVA using Log‐rank test in panels G and H, and correlation analysis was conducted in panel I

A total of 9716 DEGs were obtained through analysis of differential expression of EAC‐related genes in TCGA (Figure 2A). Among these DEGs, MYB was found to be overexpressed in EAC. Additionally, previous research has shown that c‐Myb and miR‐145‐5p are highly expressed in EAC.^13^ Through retrieval of expression of MYB in TCGA, we found that MYB showed high expression in a variety of tumors including EAC (Figure 2B). Further retrieval of MYB expression in EAC samples at different stages and in normal samples indicated that MYB expression was consistently higher in EAC samples (Figure 2C). Existing reports show that c‐Myb can promote the transcription of miR‐145.^12^ In this initial study, results revealed that c‐Myb and miR‐145‐5p expressions were higher in EAC samples than in tumor adjacent samples (Figure 2D & E). The patient groups with high and low expression of c‐Myb were divided according to the median of c‐Myb IHC scores, and survival curves were plotted. Survival curves indicated that overexpressed c‐Myb or miR‐145‐5p was associated with poor prognosis (Figure 2F & G). We normalized c‐Myb expression to that of miR‐145‐5p and undertook regression analysis, which showed a strong positive correlation between c‐Myb with miR‐145‐5p expression (Figure 2H).

### 3.3 c‐Myb activated the transcription of miR‐145‐5p by binding to miR‐145‐5p promoter

**FIGURE 3:**
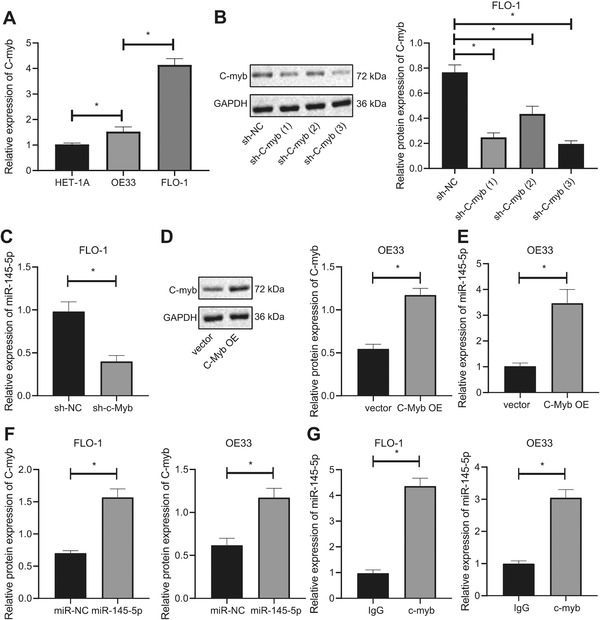
c‐Myb activates the expression of miR‐145‐5p. (A) RT‐qPCR determination of expression of c‐Myb in each EAC cell line and normal esophageal mucosal epithelial cells. (B) Western blot analysis of the expression of c‐Myb in FLO‐1 cells after treatment of sh‐c‐Myb. (C) RT‐qPCR determination of expression of miR‐145‐5p in FLO‐1 cells after treatment of sh‐c‐Myb. (D) Western blot analysis of c‐Myb expression in OE33 cells after treatment of c‐Myb OE. (E) Western blot analysis of expression in OE33 cells after treatment of c‐Myb OE. (F) The binding of miR‐145‐5p to c‐Myb in EAC cells assessed by RNA pull‐down assay. (G) The binding of miR‐145‐5p to c‐Myb in EAC cells through antibodies to IgG and c‐Myb using RIP assay. *P < .05. Measurement data were expressed as the mean ± standard deviation. One‐way ANOVA and Tukey's post‐hoc test were used for data analysis in panels A and B, and unpaired *t*‐test was employed for data in panels C‐G

qPCR results showed that EAC cell lines OE33 and FLO‐1 both had elevated c‐Myb expression compared with normal esophageal mucosal epithelial cells, where the FLO‐1 cell line showed higher c‐Myb expression compared with the OE33 cell line (Figure 3A); we used both cell lines for subsequent studies. We constructed sh‐c‐Myb to inhibit c‐Myb, and examined the inhibitory effect of each sh‐c‐Myb in the FLO‐1 cell line with high c‐Myb expression, and selected the sh‐c‐Myb sequence with the highest inhibitory efficiency (Figure 3B). Afterwards, silencing c‐Myb inhibited miR‐145‐5p expression (Figure 3C). We next constructed the c‐Myb OE sequence and results indicated that transfection of c‐Myb OE into the OE33 cell line with low c‐Myb expression increased c‐Myb expression (Figure 3D), while transfection of c‐Myb OE also increased miR‐145‐5p expression (Figure 3E). RNA pull‐down assay confirmed that miR‐145‐5p could coimmunoprecipitate c‐Myb from cell extracts both of FLO‐1 and OE33 cell lines (Figure 3F). RIP assays showed that the application of c‐Myb antibody could coimmunoprecipitate miR‐145‐5p from cell extracts of both OE33 and FLO‐1 cell lines, compared with IgG control (Figure 3G).

### 3.4 c‐Myb inhibits the activity of T cells, and increases immune escape of EAC cells by upregulating miR‐145‐5p

**FIGURE 4:**
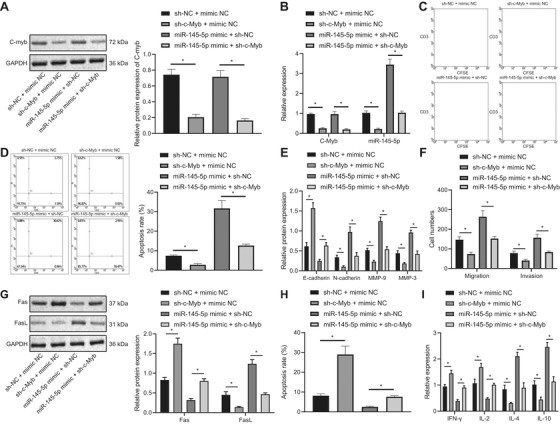
c‐Myb restrains the activity of T cells, and enhances the immune escape of FLO‐1 cell cells by stimulating the expression of miR‐145‐5p. (A) The expression of c‐Myb in each group detected using Western blot analysis. (B) Expression of c‐Myb and miR‐145‐5p in each group measured using qPCR. (C) Proliferation of T cells cocultured with different groups of FLO‐1 cells examined by CFSE dye and CD3 antibody. (D) Apoptosis of T cells cocultured with different groups of FLO‐1 cells detected using flow cytometry combining with CD3 antibody. (E) The effect of invasion and migration‐related protein expression in FLO‐1 cells of each group detected by Western blot analysis. (F) Statistics of the number of invasive and migratory cells in each group. (G) The expression of Fas and FasL in FLO‐1 cells of each group detected using Western blot analysis. (H) Apoptosis of FLO‐1 cells in different groups assessed by flow cytometry. (I) The cytokine levels secreted by T cells in each treatment group detected by ELISA. *P < .05. Measurement data were expressed as mean ± standard deviation. Data in panel C were analyzed by two‐way ANOVA, and in other panels were analyzed by one‐way ANOVA and Tukey's post‐hoc test

Subsequently, treatment with sh‐c‐Myb alone decreased expression of both c‐Myb and miR‐145‐5p in FLO‐1 cells compared to sh‐NC + mimic NC, while treatment with miR‐145‐5p mimic alone could upregulate miR‐145‐5p, whereas miR‐145‐5p expression was unchanged after miR‐145‐5p OE (Figure 4A & B). It is reported that coculture of T cells with FLO‐1 cells can simulate the recognition and inactivation of tumor cells by T cells in vitro and, thus, help to study the mechanism of tumor immune escape in vitro.^21^, ^22^ In the following experiment, we detected the proliferation and apoptosis of T cells using CFSE dye and CD3 antibody and flow cytometry combining with CD3 antibody. sh‐c‐Myb treatment could increase the cell proliferation activity of T cells and inhibit apoptosis of T cells compared with sh‐NC + mimic NC treatment, while miR‐145‐5p mimic had opposite effects; cotransfection of sh‐c‐Myb and miR‐145‐5p mimic counteracted effect of treatment with miR‐145‐5p mimic alone (Figure 4C & D).

Immune escape is associated with epithelial‐mesenchymal transition (EMT), and EMT is correlated with the invasion and migration of tumor cells.^23^ The results uncovered that transfection of sh‐c‐Myb into FLO‐1 cells reduced their invasion and migration abilities, accompanied with decreased N‐cadherin, MMP‐9, and MMP‐3 expression, and increased E‐cadherin expression, while miR‐145‐5p mimic had opposite effects; cotransfection of sh‐c‐Myb and miR‐145‐5p mimic restored effect of miR‐145‐5p mimic (Figure 4E & F). Other studies have shown that the inhibition of apoptosis caused by abnormal regulation of Fas/FasL pathway plays a role in evasion of tumor cells from immune monitoring by the organism.^24^ Therefore, we detected Fas and FasL in FLO‐1 cells as an index of the immune escape of EAC cells, and determined apoptosis of FLO‐1 cells. FLO‐1 cells transfected with sh‐c‐Myb alone had increased Fas expression and decreased FasL expression as well as increased apoptosis and that FLO‐1 cells with miR‐145‐5p OE alone had opposite effect, whereas cotransfection of sh‐c‐Myb and miR‐145‐5p mimic could counteract effect of miR‐145‐5p OE alone (Figure 4G & H). Many studies have confirmed that the secretion of Th1‐specific cytokines IL‐2, and IFN‐γ is decreased, and secretion of Th2‐specific cytokines IL‐4 and IL‐10 is increased in peripheral blood of patients with advanced tumors. Tumor growth is closely related to Th2 shift, and is positively correlated with the malignancy grade of the tumors. Indeed, there is a close relationship between Th2 dominance and immune escape of tumors.^25^, ^26^ The results unraveled that IFN‐γ and IL‐2 expression increased while IL‐4 and IL‐10 expression decreased by sh‐c‐Myb compared with sh‐NC + mimic NC, and opposite effect was achieved by miR‐145‐5p mimic alone; cotransfection of sh‐c‐Myb and miR‐145‐5p mimic could counteract effect of miR‐145‐5p mimic alone (Figure 4I).

### 3.5 miR‐145‐5p targets SPOP and inhibits its expression

**FIGURE 5:**
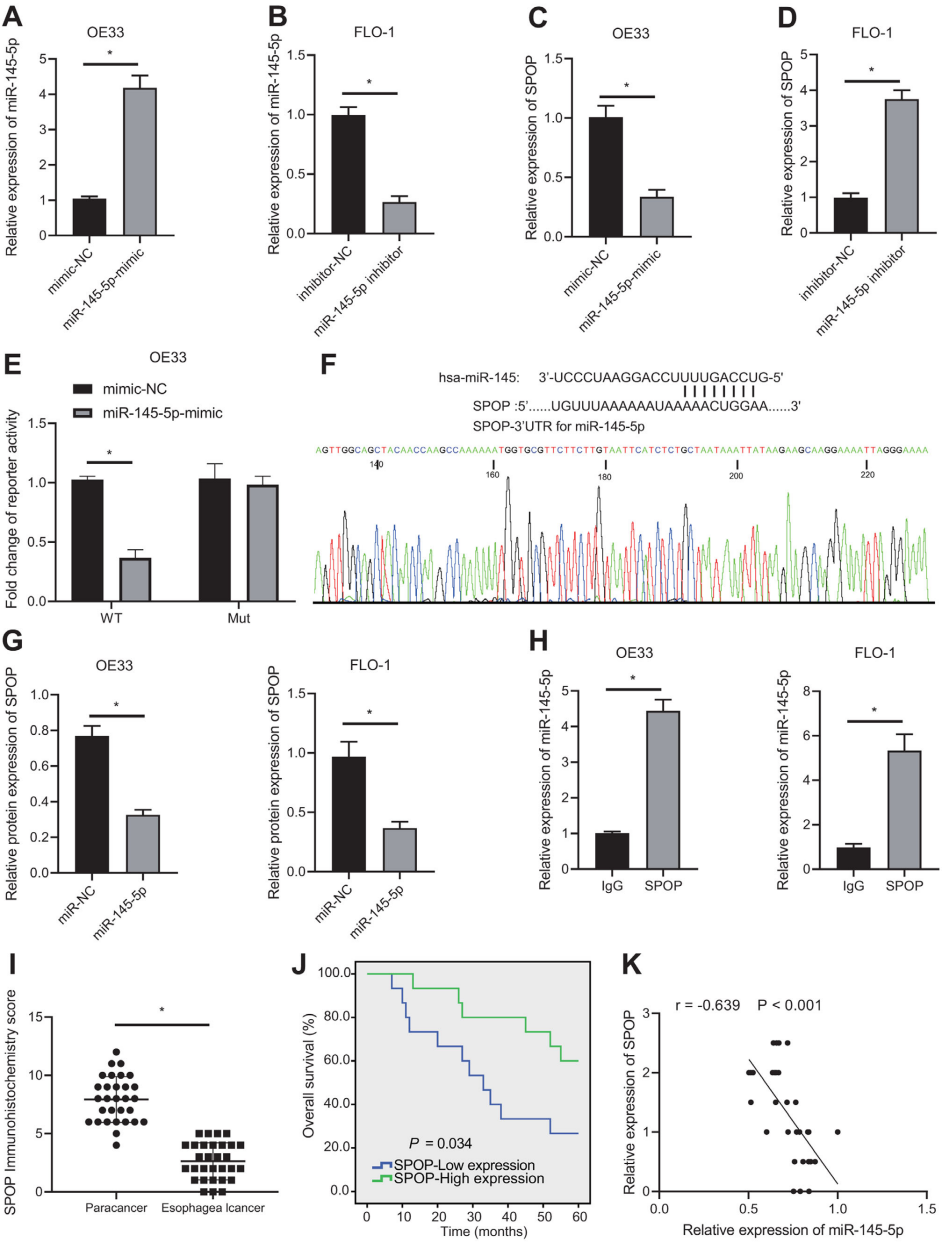
miR‐145‐5p targets SPOP. (A) Transfection effect of miR‐145‐5p mimic determined using qPCR. (B) Transfection effect of miR‐145‐5p inhibitor examined by qPCR. (C) Gene expression of SPOP after treatment of miR‐145‐5p mimic determined using qPCR. (D) Gene expression of SPOP after treatment of miR‐145‐5p inhibitor determined using qPCR. (E) Targeting relation between SPOP and miR‐145 detected using luciferase assay. (F) The binding sequence of miR‐145‐5p to the SPOP UTR detected by gene sequencing. (G) The binding of miR‐145‐5p to SPOP detected using an RNA pull‐down assay. (H) The binding of miR‐145‐5p to SPOP in EAC cells detected by IgG and SPOP antibodies using RIP assay. (I) IHC staining results of SPOP in clinical EAC tissues and tumor adjacent tissues. (J) Regression analysis of correlation of SPOP expression with overall survival in patients of EAC. (K) Correlation analysis of correlation of SPOP with miR‐145‐5p expression in EAC tissues. *P < .05. Measurement data were expressed by the mean ± standard deviation. Data in panels C‐I were analyzed by unpaired *t*‐test, and in panel J were analyzed by paired *t*‐test. Kaplan–Meier was used to calculate survival of patients followed by one‐way ANOVA using Log‐rank test in Panel I, and Pearson correlation analysis was applied for data analysis in Panel J

Most of the potential target genes had interaction relationships. qPCR results showed that miR‐145‐5p mimic increased miR‐145‐5p expression, while miR‐145‐5p inhibitor inhibited its expression (Figure 5A & B). Subsequently, miR‐145‐5p repressed SPOP expression, while inhibition of miR‐145‐5p could elevate SPOP (Figure 5C & D). Luciferase assay also demonstrated that miR‐145‐5p mimic could inhibit luciferase activity of SPOP WT (Figure 5E). We next used gene sequencing to verify binding site of miR‐145‐5p to SPOP (Figure 5F), and employed RNA pull‐down assay to confirm that miR‐145‐5p could coimmunoprecipitate SPOP (Figure 5G). RIP assay showed that the application of SPOP antibody could indeed coimmunoprecipitate miR‐145‐5p compared with IgG (Figure 5H). Further, IHC results showed that SPOP expression in EAC tissues was low (Figure 5I). According to the results of IHC scoring, the tissues with IHC score of SPOP above the median were classified into the high SPOP expression group, and those with IHC score of SPOP below the median were classified into the low SPOP expression group. Survival curve analysis showed that low SPOP expression was associated with poor prognosis (Figure 5J). Through regression analysis, we showed that SPOP had a negative correlation with miR‐145‐5p (Figure 5K).

### 3.6 miR‐145‐5p inhibits activity of T cells and increases immune escape of EAC cells via downregulation of SPOP expression

**FIGURE 6:**
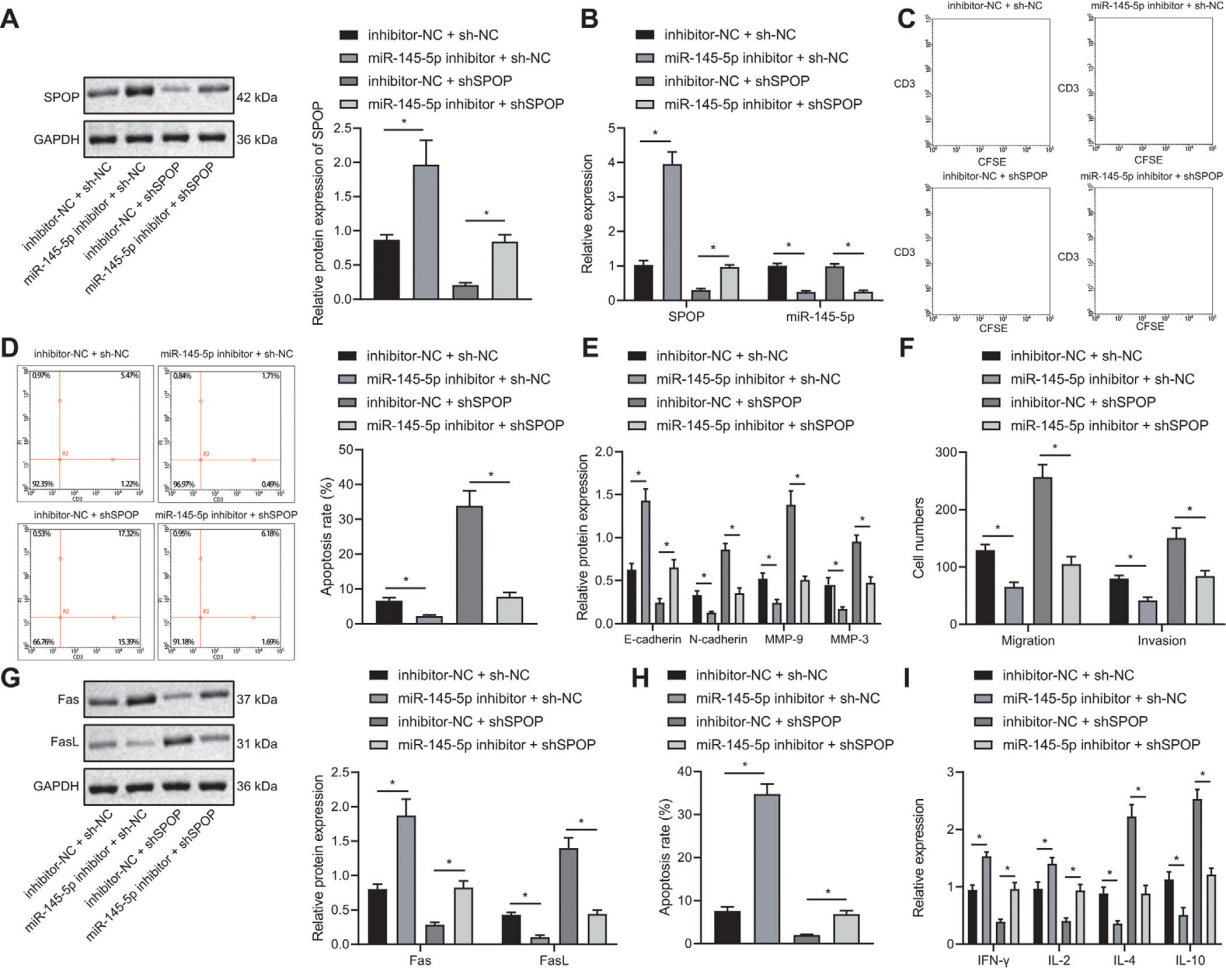
miR‐145‐5p inhibits the activity of T cells, and promotes immune escape of EAC cells (FLO‐1 cell line) by restraining the expression of SPOP. (A) The expression of SPOP in each group assessed by Western blot analysis. (B) The expression of SPOP and miR‐145‐5p in each group detected using qPCR. (C) Proliferation of T cells cocultured with different groups of FLO‐1 cells examined using CFSE dye and CD3 antibody. (D) Apoptosis of T cells cocultured with different groups of FLO‐1 cells detected using flow cytometry combining with CD3 antibody. (E) The expression of E‐cadherin, N‐ cadherin, MMP‐9, and MMP‐3 in FLO‐1 cells of each group detected by Western blot analysis. (F) The invasion and migration of different groups of FLO‐1 cells detected by Transwell assay. (G) The expression of Fas and FasL in FLO‐1 cells of each group detected using Western blot analysis. (H) Apoptosis of FLO‐1 cells in different groups detected by flow cytometry. (I) The cytokine levels secreted by T cells in each group detected by ELISA. *P < .05. Measurement data were expressed as mean ± standard deviation. Data in panel C were analyzed by two‐way ANOVA, and in other panels were analyzed by one‐way ANOVA and Tukey's post‐hoc test

SPOP expression increased and miR‐145‐5p expression decreased in FLO‐1 cells by miR‐145‐5p inhibitor alone compared with inhibitor‐NC + sh‐NC, while SPOP expression was increased by sh‐SPOP; SPOP expression was unchanged after sh‐SPOP + miR‐145‐5p inhibitor treatment (Figure 6A & B). Additionally, miR‐145‐5p inhibitor increased cell proliferation activity of T cells and inhibited apoptosis of T cells compared with treatment by inhibitor‐NC + sh‐NC, while sh‐SPOP treatment had the opposite effect; sh‐SPOP + miR‐145‐5p inhibitor could counteract effect of miR‐145‐5p inhibitor (Figure 6C & D).

miR‐145‐5p inhibitor into FLO‐1 cells could reduce their invasion and migration abilities, accompanied with decreased N‐cadherin, MMP‐9, and MMP‐3 expression, and increased E‐cadherin expression, while transfection of sh‐SPOP led to opposite effects; sh‐SPOP and miR‐145‐5p mimic cotreatment could counteract effect of miR‐145‐5p inhibitor (Figure 6E & F). Next, Fas expression increased and FasL expression decreased by miR‐145‐5p inhibitor compared with inhibitor‐NC + sh‐NC, while transfection with sh‐SPOP alone led to opposite effects; cotransfection of sh‐SPOP and miR‐145‐5p inhibitor could rescue effect of miR‐145‐5p inhibitor (Figure 6G). Moreover, miR‐145‐5p silencing exhibited enhanced apoptosis, while sh‐SPOP had decreased apoptosis compared with inhibitor‐NC + sh‐NC, but sh‐SPOP and miR‐145‐5p inhibitor cotreatment could counteract effect of miR‐145‐5p inhibitor (Figure 6H). Further, expression of IFN‐γ and IL‐2 increased while that of IL‐4 and IL‐10 decreased by miR‐145‐5p inhibitor, and an opposite effect was achieved by sh‐SPOP, while sh‐SPOP and miR‐145‐5p inhibitor cotreatment abolished effect of miR‐145‐5p inhibitor (Figure 6I).

### 3.7 SPOP promotes the ubiquitination and degradation of PD‐L1 in EAC

**FIGURE 7:**
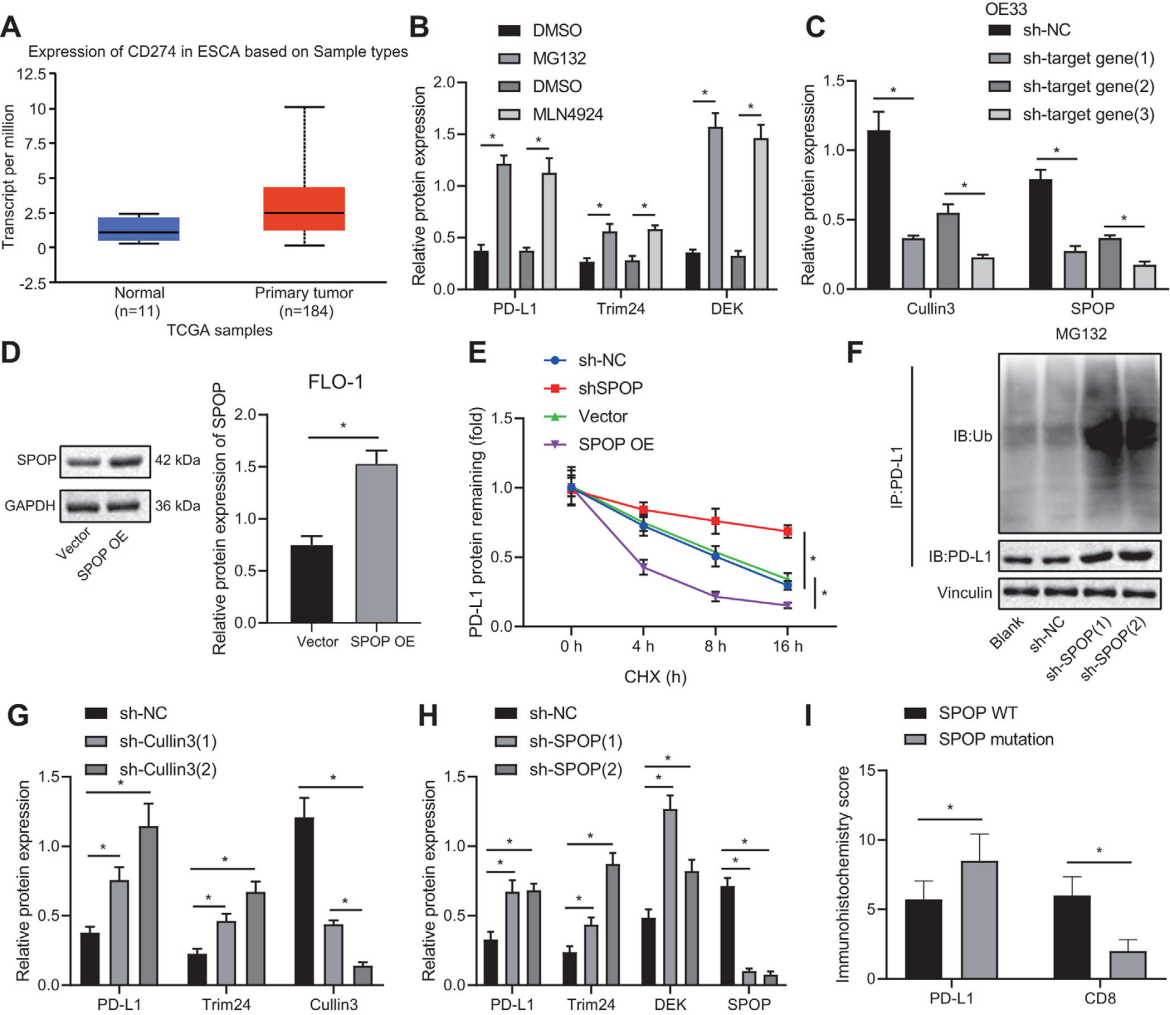
SPOP facilitates the ubiquitination and degradation of PD‐L1 in EAC. (A) The expression of CD274 (PD‐L1) gene in EAC, wherein the abscissa indicates the sample type, the ordinate indicates the expression value, the blue box plot indicates the normal sample, and the red box plot indicates the tumor sample. (B) Determination of PD‐L1, Trim24, and DEK expression by Western blot analysis after treating EAC cells with MG132 and MLN4924. (C) Construction of sh‐SPOP and shCullin3 transfection sequences and verification of their knockdown effect using Western blot. (D) Verification of the transfection effect of SPOP OE using Western blot analysis. (E) PD‐L1 expression assessed by Western blot analysis within 16 h in EAC cells transfected with sh‐SPOP or SPOP OE sequences on the basis of being treated with CHX. (F) The generation of PD‐L1‐Ub complexes detected using coimmunoprecipitation in PD‐L1 and EAC cells transfected with sh‐SPOP on the basis of being treated with MG132. (G) The expression of PD‐L1 and Trim24 examined using Western blot in EAC cells transfected with shCullin3. (H) The PD‐L1, Trim24, and DEK expression in EAC cells transfected with sh‐SPOP measured by Western blot analysis. (I) Detection of PD‐L1 and CD8 expression in the SPOP WT and SPOP mutation groups using IHC. *P < .05. Measurement data are expressed as the mean ± standard deviation. Data in panels D and J were analyzed by *t*‐test, in panel E were analyzed by two‐way ANOVA, and in panels B, C, G, H were analyzed using one‐way ANOVA and Tukey's post‐hoc test

SPOP can promote the ubiquitination modification of PD‐L1 (CD274), which in turn regulates tumor development,^16^ and that PD‐L1 gene is a DEG in the treatment of EAC.^20^ Besides, EAC‐related data in the TCGA database showed that the PD‐L1 expression is increased in EAC (Figure 7A). Furthermore, PD‐L1 can be degraded by the protease system mediated by cyclin D‐CDK4 and Cullin 3^SPOP^ E3 ligases in human cancers.^24^ However, this mechanism has not been confirmed in EAC. In this part of the study, we used the proteasome inhibitor MG132 (0.5 μM, MedChemExpress Company, #HY‐13259) and the cullin‐dependent ubiquitin E3 ligase inhibitor MLN4924 (0.09 μM, Sigma, #5054770001)^27^ to analyze the degradation pathway of PD‐L1 in EAC cells. SPOP is an adaptor molecule of the E3 ubiquitin ligase Cul3. Adaptor molecules determine the specificity of the substrate reaction, while PD‐L1, Trim24, and DEK are specific substrates of SPOP.^28^ Western blot analysis results showed that PD‐L1, Trim24, and DEK were increased in EAC cells treated with MG132 and MLN4924 in vitro (Figure 7B), indicating that proteasome inhibitors and cullin‐dependent ubiquitin E3 ligase inhibitors can both inhibit the function of SPOP and increase SPOP substrates. In the following experiments, we determined the inhibitory activities of sh‐SPOP and shCullin3 by Western blot analysis, and selected the sh‐SPOP and shCullin3 sequences with the highest silencing efficiency (Figure 7C). Then, OE33 cell lines with low c‐Myb expression were subjected to sh‐SPOP or shCullin3 transfection. The SPOP OE sequence was then constructed and its transfection effect was verified. Transfection of SPOP OE into FLO‐1 cell line with high c‐Myb expression increased SPOP expression (Figure 7D). We next used cycloheximide (CHX; 10 μM, Yeason, #40325ES03) to inhibit the endogenous protein synthesis in cells. The results presented that the PD‐L1 protein expression in the sh‐NC and vector groups was decreased within 16 h of CHX treatment. Compared with the sh‐NC group, PD‐L1 protein degradation rate in the sh‐SPOP group was decreased, while the PD‐L1 protein degradation rate in the SPOP OE group was increased in the vector group (Figure 7E). Besides, the binding of PD‐L1 to ubiquitin Ub was examined using coimmunoprecipitation, which showed that binding of PD‐L1 and Ub was reduced after treatment with sh‐SPOP (Figure 7F). PD‐L1 and Trim24 expression was noticeably increased in the absence of Cullin3 by sh‐Cullin3 (Figure 7G) and PD‐L1, Trim24, and DEK expression was also markedly increased after silencing SPOP (Figure 7H). Moreover, we detected SPOP mutations by qPCR in clinical specimens of EAC, and then divided the clinical specimens into SPOP WT and SPOP mutation groups followed by detection of PD‐L1 and CD8 expression using IHC. PD‐L1 expression was elevated, CD8 expression was reduced in SPOP mutation group compared to SPOP WT group (Figure 7I).

### 3.8 c‐Myb promotes miR‐145‐5p transcription to inhibit SPOP‐mediated ubiquitination and degradation of PD‐L1, thereby facilitating immune escape of EAC cells

**FIGURE 8:**
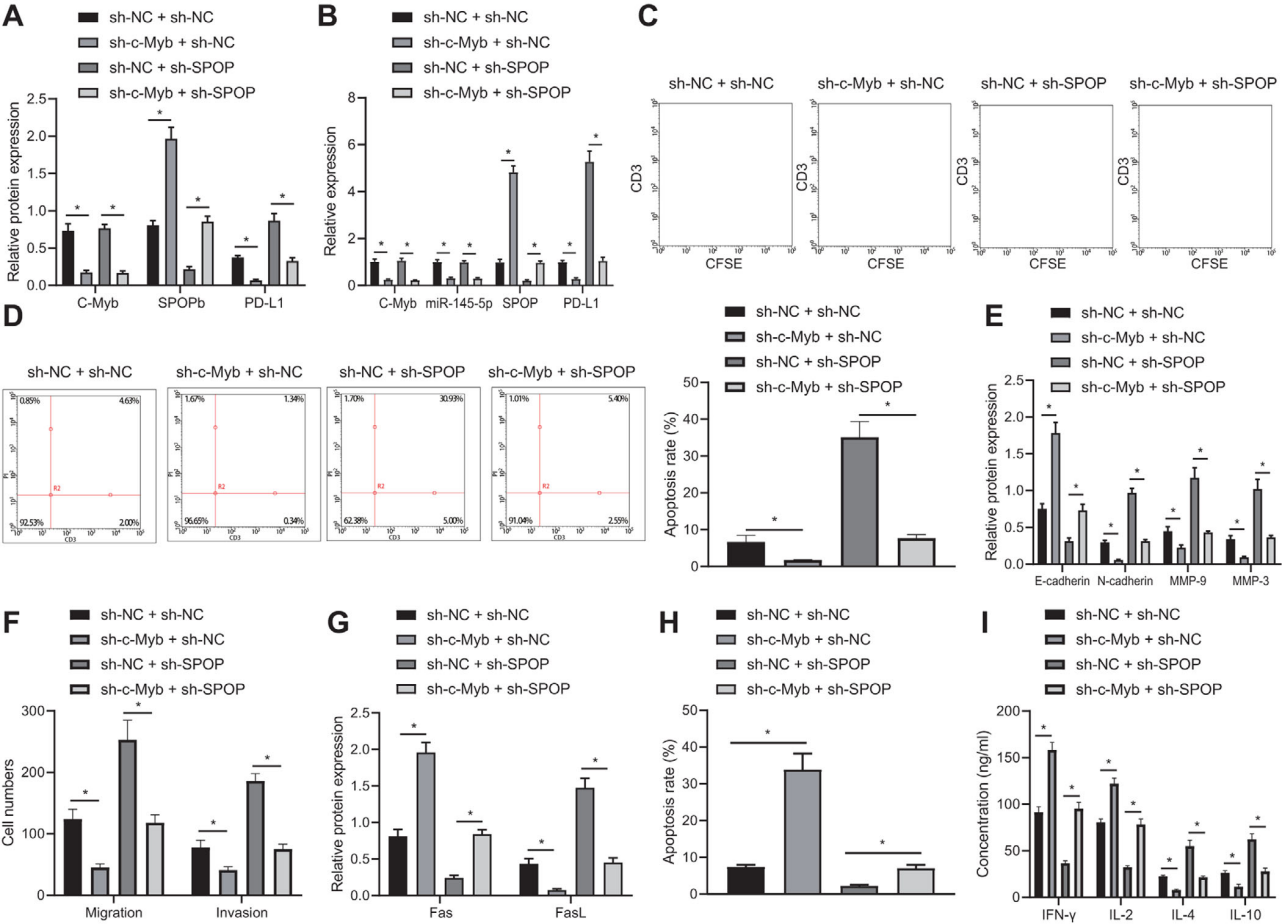
c‐Myb induces immune escape of EAC cells (FLO‐1 cell line) by promoting miR‐145‐5p transcription to inhibit the SPOP‐mediated ubiquitination and degradation of PD‐L1. (A) c‐Myb, SPOP, and PD‐L1 expression in each group assessed by Western blot analysis. (B) The expression of c‐Myb, miR‐145‐5p, SPOP, and PD‐L1 in each group detected using qPCR. (C) Proliferation of T cells cocultured with different groups of FLO‐1 cells examined using CFSE dye and CD3 antibody. (D) Apoptosis of T cells cocultured with different groups of FLO‐1 cells detected using flow cytometry combining with CD3 antibody. (E) The expression of E‐cadherin, N‐cadherin, MMP‐9, and MMP‐3 in FLO‐1 cells in each group detected by Western blot analysis. (F) The invasion and migration of different groups of FLO‐1 cells detected by Transwell assay. (G) The expression of Fas and FasL in FLO‐1 cells of each group detected using Western blot analysis. (H) Apoptosis of FLO‐1 cells in different groups assessed by flow cytometry. (I) The cytokine levels secreted by T cells in each group detected by ELISA. *P < .05. Measurement data were expressed as mean ± standard deviation. Data in panel C were analyzed by two‐way ANOVA, and in other panels were analyzed by one‐way ANOVA and Tukey's post‐hoc test

Next, c‐Myb, miR‐145‐5p, and PD‐L1 expression was decreased while SPOP expression was increased by sh‐c‐Myb alone compared with sh‐NC + sh‐NC, and that sh‐SPOP treatment increased PD‐L1 expression in FLO‐1 cells. However, PD‐L1 expression was unchanged when sh‐c‐Myb was cotransfected with sh‐SPOP (Figure 8A & B). Compared with sh‐NC + sh‐NC, treatment with sh‐c‐Myb could increase the cell proliferation activity of T cells and inhibit their apoptosis, while sh‐SPOP treatment could achieve the opposite effect. Furthermore, cotransfection of sh‐c‐Myb and sh‐SPOP could counteract the effect of treatment with sh‐SPOP alone (Figure 8C & D).

Next, sh‐c‐Myb into FLO‐1 cells reduced their invasion and migration abilities, decreased N‐cadherin, MMP‐9 and MMP‐3 expression, and increased E‐cadherin expression, while sh‐SPOP led to opposite effects; cotransfection of sh‐c‐Myb and miR‐145‐5p mimic restored effect of miR‐145‐5p mimic alone (Figure 8E & F). Fas expression increased and FasL expression decreased in EAC cells transfected with sh‐c‐Myb, while opposite effects were obtained by sh‐c‐Myb alone; cotransfection of sh‐c‐Myb and sh‐SPOP could reverse effect of sh‐SPOP treatment (Figure 8G). Since, the Fas/FasL system controls the apoptosis pathway, we examined FLO‐1 cell apoptosis. The apoptosis of FLO‐1 cells transfected with sh‐c‐Myb alone was increased, but decreased after transfected with sh‐SPOP alone compared with sh‐NC + sh‐NC. However, cotransfection of sh‐c‐Myb and sh‐SPOP could rescue the effect of treatment of sh‐SPOP (Figure 8H). Furthermore, IFN‐γ and IL‐2 expression increased while IL‐4 and IL‐10 expression decreased in the sh‐c‐Myb group, and opposite effect was achieved in the sh‐SPOP group; cotransfection of sh‐c‐Myb and sh‐SPOP could counteract the effect of treatment of sh‐SPOP (Figure 8I).

### 3.9 c‐Myb promotes the immune escape of EAC cells in vivo through upregulating miR‐145‐5p

**FIGURE 9:**
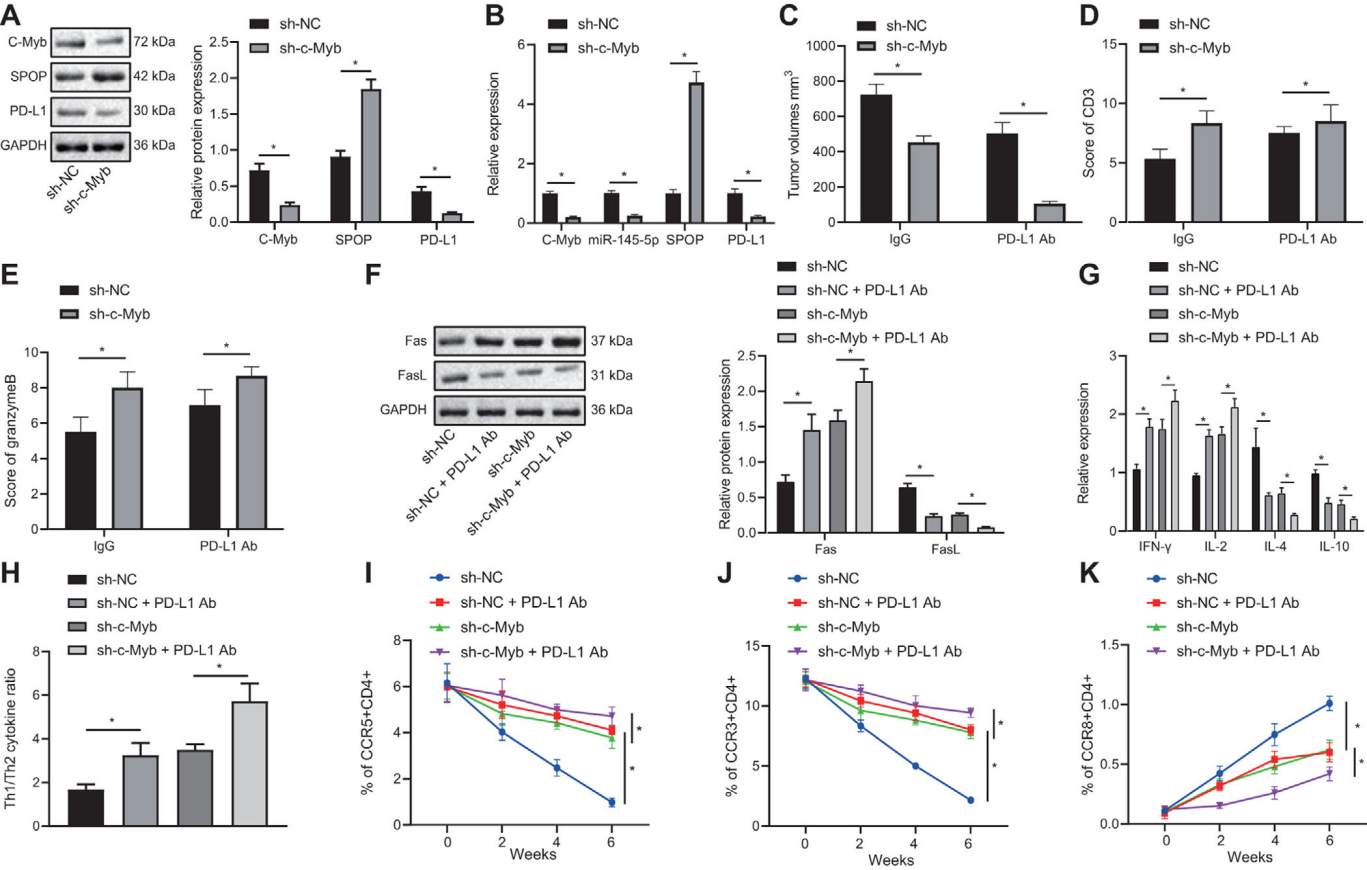
c‐Myb promotes immune escape of EAC cells (FLO‐1 cell line) by upregulating the expression of miR‐145‐5p. (A) c‐Myb, SPOP, and PD‐L1 expression in each group detected by Western blot analysis. (B) The expression of c‐Myb, miR‐145‐5p, SPOP, and PD‐L1 in each group detected using qPCR. (C) Tumor size of EAC tumor models in each group. (D) IHC diagrams of CD3 expression in EAC tissues in each group and statistical analysis of IHC scores. (E) IHC diagrams of Granzymes B expression in EAC tissues in each group and statistical analysis of IHC scores. (F) The expression of Fas and FasL in tumor tissues of each group detected by Western blot analysis. (G) The levels of cytokines secreted by T cells in each group detected using ELISA. (H) The proportion of cytokines secreted by Th1 and Th2 cells in each group detected using specific kits. (I) The proportion of CCR5 and CD4 double positive cells detected using flow cytometry. (J) The proportion of CCR3 and CD4 double positive cells assessed using flow cytometry. (K) The proportion of CCR8 and CD4 double‐positive cells assessed using flow cytometry. *P < .05. Measurement data were expressed as mean ± standard deviation. Data in panels A‐E were analyzed by unpaired *t*‐test, in panels F‐H were analyzed by one‐way ANOVA, and in panels I‐K were analyzed by two‐way ANOVA and Tukey's post‐hoc test

To validate the proposed mechanism in vivo, we constructed a mouse EAC model and injected mice with the FLO‐1 cell line transfected with sh‐c‐Myb. First, we examined c‐Myb/miR‐145‐5p/SPOP/PD‐L1 pathway proteins in tumor tissues of the model mice, which showed that c‐Myb, miR‐145‐5p, and PD‐L1 expression in the tumor tissues of model mice treated with sh‐c‐Myb was decreased, while SPOP expression was increased (Figure 9A & B). Examination of the size of tumors in each group of mice showed that sh‐c‐Myb reduced the final tumor size. Besides, h‐c‐Myb increased their therapeutic sensitivity to PD‐L1 monoclonal antibody (Figure 9C). CD3 is a protein complex that directly associates with T‐cell receptor (TCR).^29^ The cytotoxic lymphocyte protease Granzyme B can promote apoptosis by directly processing and activating caspase family members, and is one of the effector molecules of cytotoxic T cells.^30^, ^31^ By IHC analysis, we found that transfection of sh‐c‐Myb into FLO‐1 cells increased CD3 and Granzyme B expression in the tumor tissues (Figure 9D & E). sh‐c‐Myb or PD‐L1 alone into FLO‐1 cells increased Fas, IFN‐γ, and IL‐2 expression, but decreased FasL, IL‐4, and IL‐10 expression in the tumor tissues, and cotransfection of sh‐c‐Myb and PD‐L1 showed more significant trends compared with transfection of sh‐c‐Myb or PD‐L1 alone (Figure 9F & G). The proportions of cytokines secreted by Th1 and Th2 cells in the plasma of the mice were measured by specific assay kits, and the results indicated that transfection of sh‐c‐Myb or PD‐L1 alone into FLO‐1 cells increased the proportion of cytokines secreted by Th1/Th2 cells in the mice, and cotransfection of sh‐c‐Myb and PD‐L1 showed more significant trends compared with transfection of sh‐c‐Myb or PD‐L1 alone (Figure 9H). It has been reported that CXCR3 and CD4 double‐positive cells as well as CCR5 and CD4 double‐positive cells in blood can be detected by flow cytometry to express Th1 cells, and that CCR8 and CD4 double‐positive cells can express Th2 cells.^32^ Proportion of Th1 cells gradually decreased while proportion of Th2 cells gradually increased during tumor formation in the control group. However, transfection of sh‐c‐Myb or PD‐L1 alone into FLO‐1 cells attenuated the downward trend of Th1 cells and upward trend of Th2 cells, and cotransfection of sh‐c‐Myb and PD‐L1 attenuated downward trend of Th1 cells and the upward trend of Th2 cells compared with transfection of sh‐c‐Myb or PD‐L1 alone (Figure 9I‐K).

## 4 DISCUSSION

EAC is a severe health threat with increasing incidence, and it is often diagnosed at advanced stage, such that its prognosis remains dismal despite optimal treatment.^33^ Recent studies have proposed that immunotherapy could be effective for treating gastroesophageal adenocarcinoma, which is in the EAC disease spectrum.^34^ Nevertheless, tumor cells are able to employ various mechanisms to suppress tumor immunity and thereby obtain immune escape,^6^ which remains a primary challenge in immunotherapy today.^35^ Hoping to find ways to inhibit immune escape and develop more effective immunotherapeutic measures, we carried out a series of assays, which revealed that c‐Myb inhibited activity of T cells to strengthen the immune escape of EAC cells via the downstream miR‐145‐5p/SPOP/PD‐L1 pathway.

Initially, we found highly expressed c‐Myb and miR‐145‐5p in EAC, both of which markers were associated with poor prognosis of EAC, and then showed that c‐Myb could enhance miR‐145‐5p expression to increase immune escape of EAC cells through inhibiting the activity of T cells. The c‐Myb gene modulates cell growth, differentiation, and apoptosis via protein‐protein interactions and transcriptional regulation of various pathways, and is frequently overexpressed in multiple human solid tumors such as EAC.^9^, ^10^ In addition, miR‐145‐5p is reported to be highly overexpressed in EAC.^13^ Thus, c‐Myb and miR‐145‐5p may both play oncogenic roles in this cancer. Moreover, it has been proposed that MYB could facilitate the transcription of miR‐145‐5p to elevate its expression in vascular smooth muscle cells.^12^ Notably, c‐Myb plays a role in autoimmune dysfunction by participating in the development of T cells,^36^ and other work shows that high miR‐145‐5p in T cells of aplastic anemia patients attenuates the proliferation of T cells.^37^ Further, increased immune escape of tumor cells is associated with suppressed intratumor T‐cell infiltration.^38^ The above references partially supported that overexpressed c‐Myb in EAC could elevate miR‐145‐5p to induce immune escape of EAC cells through regulating T‐cell viability.

Our subsequent findings indicated that the c‐Myb/miR‐145‐5p axis further inhibits SPOP. SPOP acts as a substrate adaptor for the Cullin 3‐based ubiquitination and is involved in epigenetic control and regulation of the cell cycle, such that SPOP mutation is associated with pathogenesis of a number of cancers.^39^ For example, SPOP is likely to act as a tumor inhibitor through targeting several proteins in prostate cancer, while it may serve as an oncoprotein in renal cancer.^40^ As reported by Huang et al., miR‐145‐5p is implicated in the post‐transcriptional modulation of SPOP expression and might downregulate SPOP.^14^ Thus, it is supported to a certain degree that c‐Myb/miR‐145‐5p axis may inhibit the expression of SPOP. Moreover, our findings indicated that SPOP may promote ubiquitination and degradation of PD‐L1 to enhance immune escape of EAC cells. Consistent with this scenario, it has been reported that SPOP can inhibit tumor development by promoting the ubiquitination modification of PD‐L1.^16^ PD‐L1 plays important roles in immunosuppression and is known as a biomarker of inhibited anticancer immunity.^18^ Additionally, OE of PD‐L1 is considered as a pivotal prognostic factor for poor overall survival of EAC patients undergoing surgical treatment.^20^ It is worth noting that immunotherapy associated with PD‐1/L1 inhibition has been proposed as a treatment measure for esophagogastric adenocarcinoma.^41^ These findings suggest that knockdown of SPOP may increase PD‐L1 expression and provide support for promotive role of PD‐L1 in immune escape of EAC cells. To substantiate the findings in vitro, we also established a mouse model of EAC and confirmed in vivo that c‐Myb can enhance miR‐145‐5p and promote PD‐L1‐related immune escape of EAC cells.

## 5 CONCLUSIONS

**FIGURE 10:**
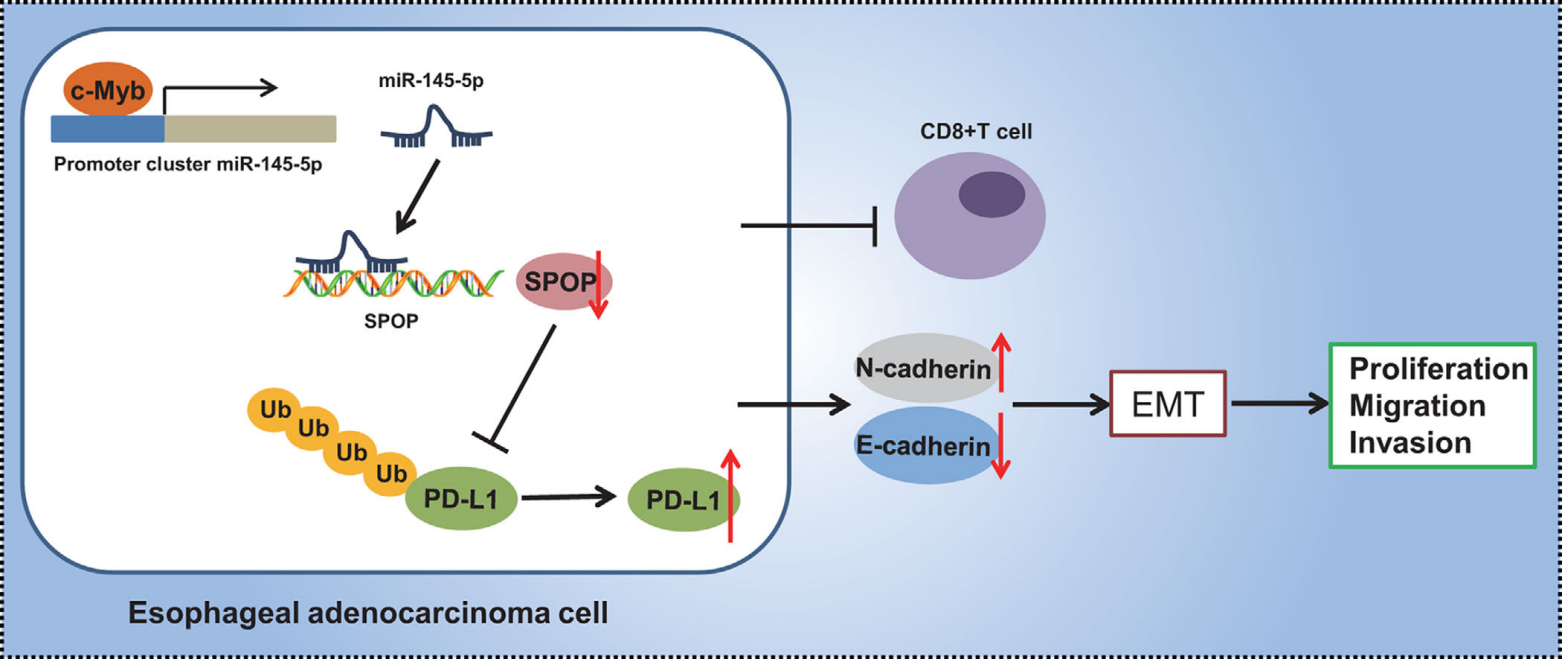
Graphical Abstract. c‐Myb can up‐regulate miR‐145‐5p expression and inhibit the SPOP‐mediated ubiquitination and degradation of PD‐L1 to strengthen of EAC cells through the SPOP/PD‐L1 pathway

To sum up, we have demonstrated in this study that c‐Myb can upregulate miR‐145‐5p expression to strengthen immune escape of EAC cells by inhibiting T‐cell viability through the SPOP/PD‐L1 pathway, a finding which sheds light on a new mechanism regarding the problematic immune escape of EAC (Figure 10). On basis of this proposed pathway, we suppose that c‐Myb and miR‐145‐5p inhibitors may help to improve the immunotherapeutic efficacy for EAC. However, this proposal needs further verification in more cell lines and clinical samples, and further studies testing the effects of c‐Myb and SPOP expression on EAC progression.

## ETHICAL APPROVAL AND CONSENT TO PARTICIPATE

All research procedures were conducted with approval of the Ethics Committee of the First Affiliated Hospital of Zhengzhou University and in line with the *Declaration of Helsinki*. All patients and/or legal guardians signed the informed consent documentation prior to experiments. All animal experiments were approved by the Animal Ethics Committee of the First Affiliated Hospital of Zhengzhou University. Great efforts were made to minimize the number of animals used in the experiments and their suffering.

## AVAILABILITY OF DATA AND MATERIAL

The datasets generated/analysed during the current study are available.

## CONFLICT OF INTEREST

The authors declare no conflict of interest.

## AUTHOR CONTRIBUTIONS

LZ and XHW designed the study. YFL, JH, and XZG collated the data, carried out data analyses, and produced the initial draft of the manuscript. SLL and FW contributed to drafting the manuscript. All authors have read and approved the final submitted manuscript.

## Supporting information

SUPPORTING INFORMATION

SUPPORTING INFORMATION

SUPPORTING INFORMATION

SUPPORTING INFORMATION
