# Supplementary material for: c‐Myb facilitates immune escape of esophageal adenocarcinoma cells through the miR‐145‐5p/SPOP/PD‐L1 axis
Source: Clin Transl Med. 2021 Sep 26;11(9):e464. doi: 10.1002/ctm2.464 (PMC8473478; doi:10.1002/ctm2.464)
Supplement: Supplementary file 1 — SUPPORTING INFORMATION [file CTM2-11-e464-s001.docx]

**Table S1** Primer sequences

| Gene | Forward | | Reverse | |
| --- | --- | --- | --- | --- |
| miR-145-5p | 5’-CAGCATACATGATTCCTTGTA-3’ | | 5’-CTTGGTGTTTGAGATGTTTGG-3’ | |
| miR-520c-3p | | GGCACAAAGTGCTTCCTTTTA | | TATGGTTTTGACGACTGTGTGAT |
| miR-520d-3p | | GGTCTACAAAGGGAAGC | | TTTGGCACTAGCACATT |
| miR-520a-3p | | GCCACCACCATCAGCCATAC | | GCACATTACTCTACTCAGAAGGG |
| U6 | 5’-TGTGTCCGTCGTGGATCTGA-3’ | | 5’-CCTGCTTCACCACCTTCTTGA-3’ | |
| PD-L1 | 5’-TGCCACCCACTGTCCTTTTA-3’ | | 5’-GTTTTCCCCTCGCATCATCC-3’ | |
| SPOP | 5’-TCTTCTGCGAGGTGAGTGTT-3’ | | 5’-CAACACACAAGCAGCAGTCT-3’ | |
| c-Myb | 5’-ACCCTGAGAAGGAAAAGCGA-3’ | | 5’-TGGAGTGGAGTGGTGTTCTC-3’ | |
| GAPDH | 5’-CTGACTTCAACAGCGACACC-3 | | 5’-CTGACTTCAACAGCGACACC-3’ | |
