# Supplementary material for: c‐Myb facilitates immune escape of esophageal adenocarcinoma cells through the miR‐145‐5p/SPOP/PD‐L1 axis
Source: Clin Transl Med. 2021 Sep 26;11(9):e464. doi: 10.1002/ctm2.464 (PMC8473478; doi:10.1002/ctm2.464)
Supplement: Supplementary file 2 — SUPPORTING INFORMATION [file CTM2-11-e464-s004.docx]

**Table S2** Plasmid sequences

| Gene | Sequences |
| --- | --- |
| miR-145-5p mimic | 5’-GUCCAGUUUUCCCAGGAAUCCCU-3’ |
| miR-145-5p inhibitor | 5’-GGAUUCCUGGGAAAACUGGACUU-3’ |
| sh-c-Myb | sc-29855-SH (SANTA CRUZ BIOTECHNOLOGY, INC.) |
| sh-SPOP | sc-63056-SH (SANTA CRUZ BIOTECHNOLOGY, INC.) |
| shCullin3 | 5’-CCGGTGGTGCTCACGACAGGATATTGTTCAAGAGACAATATCCTGTCGTGAGCACCTTTTTTGATCC-3’ |
| sh-NC | 5’-TTCTCCGAACGTGTCACGT-3’ |
